# Discussant‐Improving Dementia Detection in Primary Care: Learnings from Three Embedded, Pragmatic Clinical Trials

**DOI:** 10.1002/alz70858_101075

**Published:** 2025-12-24

**Authors:** Joseph Gaugler

**Affiliations:** ^1^ University of Minnesota, Minneapolis, MN, USA

## Abstract

**Background:**

A critical clinical and scientific goal remains the successful implementation of strategies to enhance dementia screening in those settings that older people most commonly seek help: primary care. This session highlights three innovative and highly exciting pragmatic/"real‐world" trials to determine the effectiveness and implementation potential of early detection across diverse primary care and other clinical settings.

**Method:**

I will compare and contrast how pragmatic elements were applied across the three dementia screening trials in this session and reflect upon on how these and similar efforts can advance the science of dementia care into robust Stage IV/Stage V intervention research.

**Result:**

Pragmatic trials remain an essential methodology to bridge the translational gap between traditional clinical trials and successful dissemination/implementation. The novel and innovative methods applied in this session to facilitate dementia screening in diverse primary care and similar care settings provide stellar exemplars of how pragmatic trials can expedite the dissemination and implementation of evidence‐based practices to improve dementia care.

**Conclusion:**

The use of pragmatic trial methodology can serve as a useful link between efficacy, effectiveness, and dissemination/implementation. The strengths and challenges of these three promising trials are ideal examples of how pragmatic trials can propel dementia care science to public health impact.

